# Supplementary material for: Assessing the accuracy and educational value of YouTube videos on a novel regional anesthesia technique (PENG block)
Source: PLoS One. 2026 Feb 2;21(2):e0341799. doi: 10.1371/journal.pone.0341799 (PMC12863519; doi:10.1371/journal.pone.0341799)
Supplement: Attachment — Submitted filename: Response to Reviewers.docx [file pone.0341799.s002.docx]

**Response to Reviewers**

**Manuscript ID:** PONE-D-25-59170
**Title:** *Assessing the Accuracy and Educational Value of YouTube Videos on a Novel Regional Anesthesia Technique (PENG Block)*
**Journal:** PLOS ONE

**General Response to the Editor**

Dear Academic Editor,

We thank you and the reviewers for the time and effort devoted to the evaluation of our manuscript.

All comments raised by **Reviewer #1** have been carefully addressed, and the manuscript has been revised accordingly. A detailed, point-by-point response is provided below, and all changes are highlighted in the revised manuscript with track changes.

During the revision process, we carefully reviewed the comments provided by **Reviewer #2**. We respectfully note that these comments appear to refer to a different clinical study involving patient-level data (e.g., PACU outcomes, BMI categories, Clavien–Dindo classification, critical events, and odds ratios), which are not part of our manuscript. Our study is a cross-sectional content analysis of YouTube videos related to the PENG block and does not include any clinical or patient data. We therefore believe that Reviewer #2’s comments may have been inadvertently assigned to our manuscript and kindly request your guidance on how to proceed.

All journal-specific requirements and policies have been carefully reviewed and addressed.

Kind regards,
**Muhammed Emin Zora, MD**
on behalf of all authors

**Reviewer #1**

**Comment 1**

Ensure to use the same wordings in the text as was used in the table to allow for uniformity. In the results section, regarding reference to the DISCERN scale, correct “55.6% were mild” to “55.6% were moderate.”

**Response:**
The terminology in the Results section has been revised to ensure consistency with the tables. The term “mild” has been corrected to “moderate” when referring to the DISCERN scale.

**Comment 2**

In the comparison between individuals and other sources, correct the paragraph to:
“Differences between videos from non-individual sources using metrics such as VPI, likes, comments and quality scores as compared to individual videos were not statistically significant.”

**Response:**
The Results section has been revised accordingly, and the suggested sentence has been incorporated verbatim.

**Comment 3**

In the discussion section; 1st paragraph, moderate quality does not align with poor quality.

**Response:**
The first paragraph of the Discussion section has been rephrased to align with the study findings by referring to the generally low-to-moderate or suboptimal quality of health-related YouTube content reported in previous studies.

**Comment 4**

In the last line of the 4th paragraph, it should be preferably put as:
“In future research, though using a single scale may provide an indication of overall quality, using multiple scales would enhance reliability by providing cross validation.”

**Response:**
The last sentence of the fourth paragraph in the Discussion section has been revised using the recommended wording.

**Comment 5**

The percentage figures quoted for JAMA, GQS and Modified DISCERN scales in the Results section did not tally with the table.

**Response:**
The Results section has been carefully revised to ensure full consistency between the text and Table 2. The percentage figures for JAMA, GQS, and Modified DISCERN now exactly match the tabulated data. No changes were made to the statistical analyses or tables.

**Reviewer #2**

**Response:**
The comments provided by Reviewer #2 appear to refer to a different clinical manuscript involving patient-level data and outcomes. As the present study is a YouTube-based content analysis without clinical or patient data, these comments do not apply to this manuscript. We kindly request editorial guidance regarding this issue.
